# Draft Genome Sequence of *Leuconostoc mesenteroides* 406 Isolated from the Traditional Fermented Mare Milk Airag in Tuv Aimag, Mongolia

**DOI:** 10.1128/genomeA.00166-16

**Published:** 2016-03-24

**Authors:** Hidetoshi Morita, Hidehiro Toh, Kenshiro Oshima, Akiyo Nakano, Chihiro Hano, Saki Yoshida, Tien Thi Thuy Nguyen, Wulijideligen, Kosuke Tashiro, Kensuke Arakawa, Taku Miyamoto

**Affiliations:** a Graduate School of Environmental and Life Science, Okayama University, Okayama, Japan; b Medical Institute of Bioregulation, Kyushu University, Fukuoka, Japan; c Graduate School of Frontier Sciences, The University of Tokyo, Kashiwa, Chiba, Japan; d Department of Microbiology and Infectious Diseases, Nara Medical University, Kashihara, Nara, Japan; e Department of Life Sciences, Chifeng University, Inner Mongolia, China; f Graduate School of Systems Life Sciences, Kyushu University, Fukuoka, Japan; g Faculty of Food Culture, Kurashiki Sakuyo University, Kurashiki, Japan

## Abstract

*Leuconostoc mesenteroides* 406 was isolated from the traditional fermented mare milk airag in Tuv Aimag, Mongolia. This strain produces an antilisterial bacteriocin. Here, we report the draft genome sequence of this organism.

## GENOME ANNOUNCEMENT

Airag is the most popular traditional fermented milk in Mongolia and Inner Mongolia of China. It is made by fermentation of mainly mare milk with various species of lactic acid bacteria. *Leuconostoc mesenteroides* is a species of lactic acid bacteria frequently detected in airag (1). The genus *Leuconostoc* represents a group within the low-G+C-content Gram-positive bacteria. We previously isolated *L. mesenteroides* 406, which produces an antilisterial bacteriocin, from airag in Tuv Aimag, Mongolia (2).

The *L. mesenteroides* 406 genome was paired-end sequenced using Illumina’s MiSeq platform. Genomic libraries containing 600- to 1,000-bp inserts were constructed and sequenced, yielding 3,291,624 sequences that provided 376-fold coverage from both ends of the genomic clones. The sequence reads were assembled using Newbler version 2.8 (Roche), and the assembled genome consists of 69 contigs, with a total length of 2,004,312 bp. The draft genome of strain 406 contained 1,978 predicted protein-coding genes. Next, we compared the draft genome of strain 406 with that of *L. mesenteroides* 213M0 (accession no. BCMO01000000), which was isolated from another airag in Bulgan Aimag, Mongolia (3). Of the 1,978 protein-coding genes, 1,754 genes (89%) were shared by the two strains. The genome information of this species will be useful for further studies on food science and of its physiology, taxonomy, and ecology.

### Nucleotide sequence accession numbers. 

The draft genome sequence for *L. mesenteroides* 406 has been deposited in the DDBJ/GenBank/EMBL database under the accession numbers BCMP01000001 to BCMP01000069.
